# Assessment of the Relationship between Self-Declared Ethnicity, Mitochondrial Haplogroups and Genomic Ancestry in Brazilian Individuals

**DOI:** 10.1371/journal.pone.0062005

**Published:** 2013-04-24

**Authors:** Mari M. S. G. Cardena, Ândrea Ribeiro-dos-Santos, Sidney Santos, Alfredo J. Mansur, Alexandre C. Pereira, Cintia Fridman

**Affiliations:** 1 Department of Legal Medicine, Ethics and Occupational Health, Medical School, University of São Paulo, São Paulo, São Paulo, Brazil; 2 Laboratory of Human Genetics and Medicine, Federal University of Pará, Belém, Pará, Brazil; 3 Department of Cardiology, Laboratory of Genetics and Molecular Cardiology, Heart Institute, Medical School, University of São Paulo, São Paulo, São Paulo, Brazil; Vanderbilt University Medical Center, United States of America

## Abstract

In populations that have a high degree of admixture, such as in Brazil, the sole use of ethnicity self-declaration information is not a good method for classifying individuals regarding their ethnicity. Here, we evaluate the relationship of self-declared ethnicities with genomic ancestry and mitochondrial haplogroups in 492 individuals from southeastern Brazil. Mitochondrial haplogroups were obtained by analyzing the hypervariable regions of the mitochondrial DNA (mtDNA), and the genomic ancestry was obtained using 48 autosomal insertion-deletion ancestry informative markers (AIM). Of the 492 individuals, 74.6% self-declared as White, 13.8% as Brown and 10.4% as Black. Classification of the mtDNA haplogroups showed that 46.3% had African mtDNA, and the genomic ancestry analysis showed that the main contribution was European (57.4%). When we looked at the distribution of mtDNA and genomic ancestry according to the self-declared ethnicities from 367 individuals who self-declared as White, 37.6% showed African mtDNA, and they had a high contribution of European genomic ancestry (63.3%) but also a significant contribution of African ancestry (22.2%). Of the 68 individuals who self-declared as Brown, 25% showed Amerindian mtDNA and similar contribution of European and African genomic ancestries. Of the 51 subjects who self-declared as black, 80.4% had African mtDNA, and the main contribution of genomic ancestry was African (55.6%), but they also had a significant proportion of European ancestry (32.1%). The Brazilian population had a uniform degree of Amerindian genomic ancestry, and it was only with the use of genetic markers (autosomal or mitochondrial) that we were able to capture Amerindian ancestry information. Additionally, it was possible to observe a high degree of heterogeneity in the ancestry for both types of genetic markers, which shows the high genetic admixture that is present in the Brazilian population. We suggest that in epidemiological studies, the use of these methods could provide complementary information.

## Introduction

In the past few years, various applications of ethnicity information, such as in forensic science, epidemiological studies, and clinical and pharmacological trials, have been proposed in the literature. However, in highly admixed populations, such as in Brazil, this personal information cannot provide the same robust estimations as in less diverse populations [1]–[3].

The Brazilian population is one of the most heterogeneous in the world and is the result of five centuries of crossings between inter-ethnic individuals from three continents, European settlers, African slaves and Brazilian native Amerindians [4], which results in the incorporation of various social cultures in Brazil. Consequently, the genotypic and phenotypic characteristics of these populations have been added to the native population [5], [6]. This high rate of admixture makes physical appearance characteristics such as skin and eye color, hair, and the shape of the lips and nose poor indicators of the geographical origin of a Brazilian individual's ancestors [7].

Ancestry Informative Markers (AIMs) are autosomal markers that have been used to estimate the genomic ancestry of a population or individual because they show differences in allele frequencies between distinct populations [8]–[10]. These markers have a substantial advantage with respect to physical features because they are constant throughout life [7], [11].

Mitochondrial DNA (mtDNA) has also proved to be a good marker for inferring maternal ethnicity. Several studies have indicated the feasibility of inferring the probable geographic origin of an individual from the sequence of hypervariable regions (HV) of the mitochondrial genome. These studies clearly demonstrate that the mitochondrial sequence alone does not determine one's ethnicity because it relates exclusively to maternal inheritance [2], [12], [13].

Therefore, to understand the relationship between the population sub-structure and the genetic makeup, autosomal markers are routinely used to estimate individual ancestry, whereas markers in the mtDNA and Y chromosome are used to scale inferences about human evolutionary history. The analysis of both types of information, AIMs and mtDNA, could be strongly associated and, thereby, used to infer more accurately the ethnic origin of an individual [2], [12], [13].

The aim of this study was to evaluate the relationship between self-declared ethnicity, genomic ancestry and mitochondrial haplogroups (mtDNA) in 492 individuals from southeastern Brazil.

## Materials and Methods

### Population Samples

We studied 492 individuals who had volunteered as part of a healthcare program developed by the Heart Institute of the Medical School, University of São Paulo, located in the Southeast of Brazil (São Paulo, SP-Brazil). The volunteers answered a questionnaire that included a multiple-choice question on self-declared ethnicity, which was based on the method used by the Brazilian Institute of Geography and Statistics (IBGE) national census survey, which classifies individuals as “Brancos” (i.e., White), “Pardos” (i.e., Brown), “Pretos” (i.e., Black), “Amarelos” (i.e., Yellow) and “Indígenas” (i.e., Indigenous).

All of the individuals signed an informed consent form, and the Ethics Committee of the Clinical Hospital from the Medical School, University of São Paulo, approved the research protocol. The individuals were included in the study from August 2002 to March 2004.

### Mitochondrial DNA Analysis

DNA was extracted from peripheral blood leukocytes following standard salting out techniques [14].

The samples were analyzed for the hypervariable regions HV1, HV2 and HV3 to obtain the mtDNA composition of the haplotypes (sequences) and their mtDNA classification.

The DNA samples were amplified by a single PCR reaction, using primers designed with the Primer3 program (http://www-genome.wi.mit.edu/cgi-bin/primer/primer3_www.cgi). The primers used were L15879 (5′-AATGGGCCTGTCCTTGTAGT-3 ′) and H727 (5′-AGGGTGAACTCACTGGAACG-3′). The amplified segment refers to the nucleotide sequence 15879–727, which contains 1417 base pairs (bp) comprising the three regions of interest (HV1, HV2 and HV3).

The PCR reactions were performed in a total volume of 10 µl that contained 50 ng of genomic DNA, 2.5 pMol of each primer (Invitrogen Life Technologies, Carlsbad, CA), 1.25 mM dNTP (Invitrogen Life Technologies, Carlsbad, CA) and 0.75 units of Platinum Taq polymerase (Invitrogen Life Technologies, Carlsbad, CA). Thermocycling was performed in a thermocycler Eppendorf Mastercycler under conditions of initial denaturation at 95°C for 1 minute followed by 36 cycles of denaturation at 95°C for 1 minute and annealing at 60°C for 1 minute and extension 72°C for 1 minute. The final extension was at 72°C for 7 minutes.

The PCR products were purified using Exonuclease I and Shrimp Alkaline Phosphatase (EXO/SAP, Thermo Scientific Fermentas), according to the manufacturer's recommendations, and were sequenced using the BigDye Terminator v3.1 Cycle Sequencing Kit (Applied Biosystems, Foster City, CA) according to manufacturer's protocol. All of the samples were sequenced in both directions, forward and reverse, using the same PCR primers, L15879 and H727, respectively. For the samples that showed length heteroplasmy in the HV1 or HV2 regions, additional primers L16548 (5′-GGGAACGTGTGGGCTATTTA-3′) and H16413 (5′-TGAAATCAATATCCCGCACA-3′) were used in a new sequencing reaction to analyze all of the sequence. Capillary electrophoresis was performed in an automated sequencer ABI3130 (Applied Biosystems, Foster City, CA), and the results were analyzed using specific software called BioEdit (http://www.mbio.ncsu.edu/bioedit/bioedit.html).

Individual sequences were compared with the Cambridge Reference sequence (rCRS) [15], [16] using the ClustalW alignment program (http://www.ebi.ac.uk/Tools/msa/clustalw2/) and the program Haplosite (http://www.haplosite.com/haplosearch/) for final definition of the haplotypes. The differences found in each sequence regarding the rCRS were typed following the nomenclature recommendations [17]–[21]. Classification of mtDNA was performed using specific programs, mtDNAmanager (http://mtmanager.yonsei.ac.kr/search_sample.php) and Phylotree (http://www.phylotree.org/tree/main.htm) [22].

### Ancestry Informative Marker Analysis

The evaluation of genomic ancestry was conducted using forty-eight biallelic ancestry informative markers (AIMs), type insertion-deletion (INDELS), from autosomal chromosomes, which were pre-selected based on three main criteria: (1) large differences in the allelic frequencies (δ≥40%) between African, European and Native American populations, (2) mapping on different chromosomes or in different regions of the same physical chromosome, and (3) variable size between 3 and 40 bp to allow simultaneous genotyping of multiple markers. The selection process was based on data from Weber et al. [23] and the online database from the Marshfield Clinic (http://www.marshfieldclinic.org/mgs/?page=didp).

For this study, we selected 16 markers of African ancestry, 16 markers of European ancestry and 16 Native American ancestry-informative markers, presenting high values of δ between Africans versus Europeans or Native Americans. They were previously used by Santos et al. [24] and have been successfully used to assess the parental genetic contribution in different populations [25]–[27]. All of the DNA markers used were ascertained to be sensitive for indicating bio-geographic ancestry on the level of the three continental regions (Africa, Europe and Native American) that are expected to have contributed to the current Brazilian population.

The DNA samples were genotyped for 48 INDELS using three amplifications of type 16-plex PCR. All of the multiplex PCR was performed in a final volume of 12.5 µL that contained 10 ng of DNA, 10 µMol forward and reverse primers and 60 μL of Taq PCR Master Mix (Qiagen). The conditions for thermocycling PCR were 11 min at 95°C followed by 1 min at 94°C; 1 min at 60°C and 2 min at 70°C for 10 cycles; followed by 1 min at 90°C, 1 min at 60°C and 2 min at 70°C for 17 cycles; and a final extension of 60 min at 60°C.

Before capillary electrophoresis, 1 µL of the PCR product was added to a mix that contained 8.7 µL of deionized formamide HI-DI (Applied Biosystems, Foster City, CA) and 0.3 µL GeneScan 500 LIZ size standard (Applied Biosystems). DNA fragments were separated using an ABI 3130 Genetic Analyzer (Applied Biosystems), and the results were analyzed with GeneMapper IDv3.2 software (Applied Biosystems).

Estimation of the parental ancestry of the Brazilian samples was performed using the software STRUCTURE v.2.2 (http://pritch.bsd.uchicago.edu/software.html) considering three parental populations (Native American, European, and African) from our database, which was evaluated by Santos et al. [24] and Francez et al. [26].

## Results

Our sample was composed of 297 (60.4%) men and 195 (39.6%) women. The mean age of the individuals was 58 years (standard deviation (SD) ±14.4) (minimum 18; maximum 93; median 59). From a total of 492 individuals evaluated in this study, 74.6% self-declared as white, 13.8% as Brown and 10.4% as Black (Table 1).

**Table 1:** Characterization of the 492 Brazilian individuals.

Variables		Results
Age (years), n(mean ± SD)		492 (58±14.4)
Gender, n (%)	Male	297 (60.4)
	Female	195 (39.6)
Self-Reported Ethnicities, n (%)	White	367 (74.6)
	Brown	68 (13.8)
	Black	51 (10.4)
	Yellow	5 (1.0)
	Indigenous	1 (0.2)

Of the 492 individuals, 46.3% (n = 228) showed African mtDNA; the remainder were almost evenly distributed between Amerindian and European mtDNA, 28.7% (n = 141) and 25% (n = 123), respectively. From the analysis of 48 INDELs, the major contribution in our sample was of European genomic ancestry, with an average of 57.4% (±22.1%), followed by the contribution of African genomic ancestry at 28.3% (±21.7%) (Table 2).

**Table 2:** Distribution of mtDNA classification and genomic ancestry according to the self-declared ethnicities.

Variables		Total (n = 492)	Self-Reported Ethnicities				
			White (n = 367)	Brown (n = 68)	Black (n = 51)	Yellow (n = 5)	Indigenous (n = 1)
**mt-DNA, n (%)**	**African**	228 (46.3)	138 (37.6)	47 (69.1)	41 (80.4)	1 (20.0)	1 (100)
	**Amerindian**	141 (28.7)	116 (31.6)	17 (25.0)	6 (11.8)	2 (40.0)	-
	**European**	123 (25.0)	113 (30.8)	4 (5.9)	4 (7.8)	2 (40.0)	-
**Genomic Ancestry, mean ± SD**	**African**	0.283±0.217	0.220±0.169	0.410±0.212	0.556±0.236	0.204±0.154	0.562
	**European**	0.574±0.221	0.633±0.190	0.454±0.205	0.321±0.211	0.557±0.193	0.404
	**Amerindian**	0.143±0.101	0.147±0.101	0.136±0.106	0.113±0.077	0.239±0.159	0.034

When determining the distribution of mtDNA haplogroups according to self-declared ethnicities, it is interesting to note that of 367 individuals who self-declared as white, most of them, 37.6% (n = 138), showed African mtDNA, with the remaining individuals being distributed equally between Amerindian and European mtDNA, 31.6% (n = 116) and 30.8% (n = 113), respectively. Of the individuals who self-declared as black and brown, 80.4% (n = 41) and 69.1% (n = 47), respectively, had African mtDNA. Interestingly, 25% (n = 17) of the individuals who self-declared as brown showed Amerindian mtDNA. The individuals who self-declared as yellow had Amerindian and European mtDNA, with 40% (n = 2) and 40% (n = 2), respectively. The individual who self-declared as indigenous had African mtDNA (Table 2).

The genomic ancestry results showed that individuals who self-declared as White had a higher contribution of European ancestry (63.3%±19%), but we must emphasize that these individuals also had a significant contribution from African genomic ancestry (22.2%±16.9%). Individuals who self-declared as brown showed similar average contribution of European (45.4%±20.5%) and African (41%±21.2%) genomic ancestries. Of those who self-declared as black, the main genomic contribution was from African genomic ancestry (55.6%±23.6%), but these individuals also had a significant proportion of European genomic ancestry (32.1%±21.1%). For those who self-declared as yellow, the main contribution was from European genomic ancestry (55.7%±19.3%), and for the individual who self-declared as indigenous, the main contribution was from African genomic ancestry (56.2%) followed by European genomic ancestry (40.4%) (Table 2).

**Figure 1:**
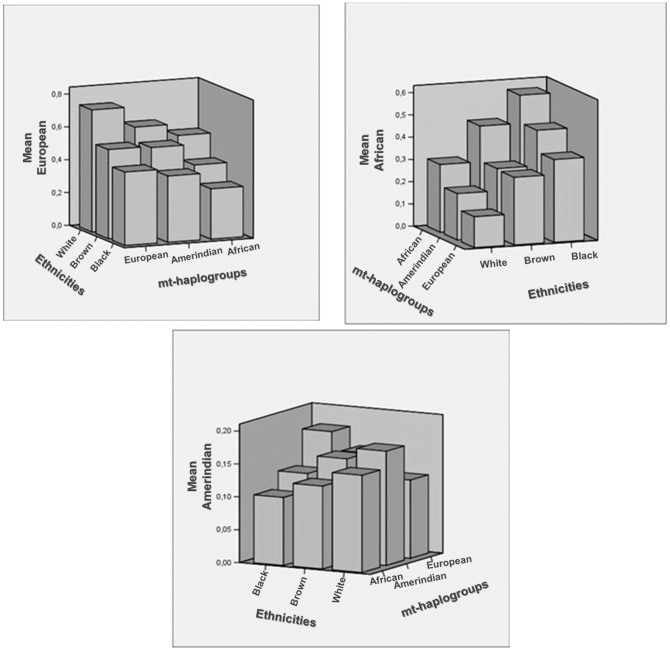
Distribution of each genomic ancestry in relation to mtDNA and the self-declared ethnicities.

When analyzing the three pieces of information together (self-declared ethnicities, mtDNA and genomic ancestry), it was possible to observe that the contribution of European genomic ancestry was higher in European mtDNA for individuals who self-declared as white and was lowest in African mtDNA for individuals who self-declared as black (Figure 1). The major contribution of African genomic ancestry was more prevalent in African mtDNA, in black and brown subjects, and in Amerindian mtDNA in black individuals (Figure 1). Interestingly, the Amerindian contribution had the same distribution in all of the self-declared ethnicities and mtDNA (Figure 1). Nonetheless, the full spectrum of combinations was observed in our sample.

Individual results can be analyzed in Table S1.

## Discussion and Conclusions

Of the 492 individuals who reported their ethnicity, 74.6% self-declared as white (Table 1), which suggests a social process of “whitening”. According to the Institute of Geography and Statistics (IBGE), Brazil's population is composed of 48.2% individuals who self-declare as white, and in southeastern Brazil, this percentage is 56.7% [28]. Currently, the demographic censuses are the only source of nationwide information on the ethnic composition of the Brazilian population. However, there are doubts about the information that is generated by the self-definition of skin color, mainly because of two considerations. The first consideration arises from the large number of terms that Brazilians use to identify the variations in skin color between the two extremes (white and black). The second consideration refers to the influence of other variables on the ethnic classification, such as social position, the subjective perception of their color, education, sex, the age of respondents, and regional and cultural variations [29], [30].

Moreover, even in Brazil, for some people there is still the concept that black individuals, i.e., people with dark skin, are related to many types of negative stereotypes, lower social class and social exclusion. In this context, the individuals who are brown or even black and who have lighter skin have a tendency to self-declare as white [31]. Government programs aimed at social inclusion use self-declared ethnicity as inclusion/exclusion criteria, making ethnic self-declaration an even more complex construct to be dissected.

Furthermore, a recent study by Leite et al. [32] demonstrated that even among biological brothers, there is disagreement in the declaration of ethnicity: in this study, one brother self-declared as white and another as brown. These findings demonstrate the existence of problems in the methodology that is used for acquiring information on ethnicity in the Brazilian population, which results in major difficulties when using these data in epidemiological studies. Various genetics studies with different Brazilian sub-populations have demonstrated the discrepancy between the self-declared information and the individual's genetic background [6], [32], [33], including the present study.

Regarding mitochondrial analysis, our results showed that 46.3% (n = 228) of all of the individuals and 37.6% (n = 138) of the individuals who self-reported as white presented African mtDNA (Table 2). This result is quite similar to Alves-Silva et al. [4], which assessed 247 individuals who self-declared as white from five major geographic regions of Brazil. When evaluating only the 99 individuals from the southeast (especially from the state of Minas Gerais), they found 34% with African mtDNA, 33% with Amerindian mtDNA and 31% with European mtDNA [4]. Clearly, the self-definition of skin color is not completely related to matrilineal ancestry in the southeastern Brazilian population.

When comparing the mtDNA in each of the three ethnicities, we observed that there were disparities: in individuals who self-declared as white, 37.6% (n = 138) had African mtDNA, while 25% (n = 17) of the individuals who self-declared as brown had Amerindian mtDNA (Table 2). These data emphasize the fact that in a population where there is a high degree of admixture and where superficial physical traits can vary with age and environmental factors, using only the self-declaration of ethnicity is not a good method for ethnic classification [7], [32], [33].

Human ethnic identity is a difficult and complicated topic. Every human being is a complex mosaic of genetic material from multiple sources of ancestors. Despite this complexity, humans can be divided in a general way into different ethnic groups that typically reflect their geographic ancestry, by using uni-parental and/or bi-parental markers [2]. Because of this, some studies have suggested that mitochondrial DNA can be an excellent marker for inferring maternal ethnic affiliation. Although the mtDNA represents only a very small segment of the mosaic of the genetic ancestry of a human being, it also provides reliable information about the range of composition and the proportions of an individual's ancestral roots, and this information could be very useful in clinical and forensic investigations [2], [13].

The genomic ancestry found in this study showed that the Brazilian population has a major contribution of European ancestry (57.5%±22.2%) (Table 2). Although these data corroborate with previous studies in relation to the major contributions of genomic ancestry [3], [34], it is possible to observe that there were differences between our data and these other studies because we found a slightly larger percentage of African genomic ancestry and a slightly lower percentage of European genomic ancestry in the black and brown individuals. Differences regarding the described mean ancestry of the studied individuals can be mainly due to the socio-economic bias that exists in the Brazilian population. The Pena et al. sample was obtained in a high socio-economic class (undergraduate and graduate students and civil servants), while our sample was from individuals with heart failure who were being followed in a public hospital. Interestingly, the Amerindian genomic ancestry component tends to be rather low in our study and in previous studies [3], [32]–[34].

When analyzing the genomic ancestry contributions in each of the three ethnicities that have been reported, it is interesting to note that there is considerable admixture of the three ancestries in all of the ethnic groups (Table 2). Moreover, we could observe what was expected, namely, that individuals who reported being white had, on average, a greater European contribution than individuals who reported being brown, and the lowest European contribution was observed among those who reported to be black. The opposite is observed for the African contribution. These observations in our study were also observed in other studies on the Brazilian population [7], [32], [33].

An important point to highlight is that only with the use of genetic markers (autosomal or mitochondrial) were we able to capture the information about the Amerindian component of the Brazilian population, even with only one individual who was self-declared as indigenous. We observed a 14.2% (±10%) genomic contribution from Amerindian ancestry, and 28.7% (n = 141) of the subjects had Amerindian mtDNA when we looked at the sample as a whole (Table 2).

By combining the ancestry information of self-declared ethnicities, matrilineal and bi-parental markers, it is possible to observe a high degree of heterogeneity of the ancestry in both types of genetic markers (mtDNA and AIMs), which shows the genetic mix in the Brazilian population; these results cannot be measured by using only self-declared ethnicity (Figure 1, Table S1). Again, the combined results show that, in Brazil, the self-defined ethnicity has relatively little relationship to the genetic ancestry of each individual, a fact that has been demonstrated and discussed by other studies on the Brazilian population [3], [7], [10], [32]–[34].

Although these studies have demonstrated that there is no strong correlation between self-declared ethnicity and genetic ancestry, in the present study, we suggest that increased information content can be brought through the use of combined information from self-declared ethnicity with the different types of inheritance, uni-parental and bi-parental.

By using mtDNA, which provides a clear pattern of historical events that are not obscured by the factors of recombination [35], and by using information from AIMs, which can estimate individual and population interethnic admixtures [8], one can have an accurate reconstitution of the genetic ancestry of a certain population or individual. Nevertheless, self-declared ethnicity can bring different information to the researcher. This self-declared information is the result of visual traits such as skin color combined with socio-cultural aspects [36], [37] and is determined by factors that might not be captured by genetic markers of admixture or geographical ancestry.

Thus, the results of this study emphasize that it is important to understand that genetic ancestry information provides more accurate estimates of the continental ancestry of an individual, while the self-reported ethnicity indirectly provides important information about his/her socio-economic and cultural background. Thus, it is tempting to suggest that the combined use of this information can provide new and complementary insights in the understanding of complex phenotype determination.

There are potential limitations to our study. First, we do not have information on Y-chromosome markers. Additionally, by using patrilineal information, one can anticipate that more information would be available, and most likely a different covariance structure between the described dimensions would arise. Second, we have not considered East Asian ancestry markers in our model. This specific ancestry is most likely biasing Native-American components. Nonetheless, we do not believe that the number of individuals of Asian descent in the studied sample significantly biases our results.

We demonstrated that the genetic ancestry varies significantly between different self-declarations of skin color as well as between the same ethnic groups. Moreover, the image of complex genetic ancestry detected in our study highlights the need for the use of combined information from self-reported ethnicity with sensitive markers of both uni-parental, as well as bi-parental ancestry, to obtain more precise conclusions about bio-geographical origin, which is relevant in epidemiological studies and in historical and forensic areas.

In conclusion, this study revealed that the genomes of most Brazilians are mixed, having mtDNA and genomic ancestry of different phylogeographical origins, and that these markers are also capable of providing new and valuable insight into the current structure of the Brazilian people. Regardless of their skin color, the majority of individuals from southeastern Brazil presented African matrilineages; they have a high degree of European genomic ancestry and a very uniform degree of Amerindian genomic ancestry. Because of the overall low correlation between ethnic, genetic and matrilineal information, we propose that, especially in admixed populations, the use of a three-dimensional construct can provide more predictive power when studying the association between ethnicity and complex human phenotypes.

## Supporting Information

Table S1 **Individual Results of Self-declared, AIMs analyses, Mitochondrial Haplogroups and Mitocondrial DNA Haplotypes.**
(XLS)
